# Mechanical Capsid Maturation Facilitates the Resolution of Conflicting Requirements for Herpesvirus Assembly

**DOI:** 10.1128/jvi.01831-21

**Published:** 2022-02-23

**Authors:** Alex Evilevitch, Udom Sae-Ueng

**Affiliations:** a Department of Experimental Medical Science, Lund University, Lund, Sweden; b Department of Physics, Carnegie Mellon University, Pittsburgh, Pennsylvania, USA; University of Toronto

**Keywords:** HSV-1, UL25, atomic force microscopy, capsid, mechanical maturation, stability

## Abstract

Most viruses undergo a maturation process from a weakly self-assembled, noninfectious particle to a stable, infectious virion. For herpesviruses, this maturation process resolves several conflicting requirements: (i) assembly must be driven by weak, reversible interactions between viral particle subunits to reduce errors and minimize the energy of self-assembly, and (ii) the viral particle must be stable enough to withstand tens of atmospheres of DNA pressure resulting from its strong confinement in the capsid. With herpes simplex virus 1 (HSV-1) as a prototype of human herpesviruses, we demonstrated that this mechanical capsid maturation is mainly facilitated through capsid binding auxiliary protein UL25, orthologs of which are present in all herpesviruses. Through genetic manipulation of UL25 mutants of HSV-1 combined with the interrogation of capsid mechanics with atomic force microscopy nano-indentation, we suggested the mechanism of stepwise binding of distinct UL25 domains correlated with capsid maturation and DNA packaging. These findings demonstrate another paradigm of viruses as elegantly programmed nano-machines where an intimate relationship between mechanical and genetic information is preserved in UL25 architecture.

**IMPORTANCE** The minor capsid protein UL25 plays a critical role in the mechanical maturation of the HSV-1 capsid during virus assembly and is required for stable DNA packaging. We modulated the UL25 capsid interactions by genetically deleting different UL25 regions and quantifying the effect on mechanical capsid stability using an atomic force microscopy (AFM) nanoindentation approach. This approach revealed how UL25 regions reinforced the herpesvirus capsid to stably package and retain pressurized DNA. Our data suggest a mechanism of stepwise binding of two main UL25 domains timed with DNA packaging.

## INTRODUCTION

Virion maturation and assembly are driven by both intermolecular interactions and mechanical forces (1, 2). Large mechanical forces are associated with the process of viral DNA packaging in herpesviruses (3, 4), which requires the assembly of a robust capsid capable of retaining the DNA. However, the maturation mechanism leading to a mechanically stable capsid remains poorly understood (5). This study presents a striking demonstration of how genetic and mechanical information within viral capsid proteins is intimately connected to ensure successful stabilization of herpesvirus capsids that are likely timed with encapsidation of the viral genome. We used herpes simplex virus 1 (HSV-1) as an experimental model system of human herpesviruses.

Herpesviruses present an intriguing nano-mechanical system. They package their micrometer-long dsDNA (125 to 230 kbp depending on the type of herpesvirus) into a nanometer-scale spherical icosahedral capsid with the help of a viral ATPase motor located at one unique capsid vertex. Fully packaged intracapsid DNA exerts tens of atmospheres of mechanical pressure on the capsid interior walls (4). This capsid pressure ejects DNA into a host cell nucleus, triggering viral infection (6). While stabilized herpesvirus capsid is required to withstand high internal DNA pressure (4, 7), one of the general principles of viral capsid assembly is the formation of weak bonds between capsid subunits. This allows for an error-free assembly with minimized free energy (8, 9). We previously found that herpesviruses resolve these contradicting requirements through a mechanical capsid maturation process (3). During HSV-1 assembly, a spherical procapsid is first formed with weaker capsomer-capsomer interactions (10–13). Once DNA packaging is initiated, the procapsid undergoes several maturation steps that involve structural transitions and binding of minor capsid proteins, resulting in a stable capsid that constitutes an infectious virion (13). Specifically, the binding of protein UL25 to the capsid surface strongly reinforces the capsid structure. We hypothesized that this step facilitates stable DNA packaging (3, 14). Despite decades of study (10, 15–28), the details of UL25’s interactions with the capsid are not fully understood due to its complex multiprotein capsid attachment and flexible regions, which reduce the imaging resolution (29). This knowledge is critically important for understanding UL25’s functionality in the herpesvirus maturation process.

**FIG 1:**
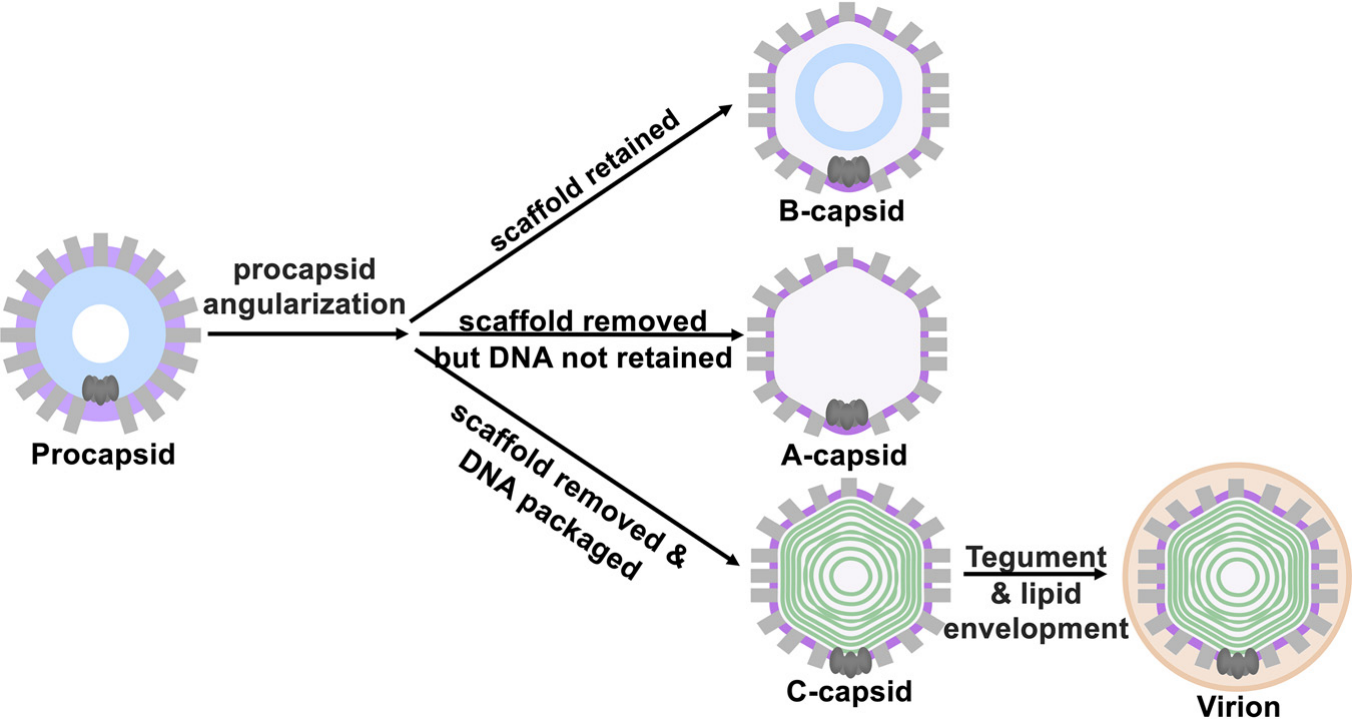
HSV-1 capsid assembly starts with the cleavage of the scaffold and angularization of the spherical, instable procapsids into three stable capsid types resulting from a mismatch in timing between scaffold digestion and DNA packaging: scaffold-containing B-capsids; empty A-capsids, which initiated the DNA packaging process but failed to retain the genome; and DNA-filled C-capsids. Only C-capsids turn into infectious virions after recruitment of tegument proteins and lipid envelopment.

During HSV-1 assembly, spherical procapsid is formed around a scaffold protein (10, 11, 30). During DNA packaging the scaffold is cleaved and removed, yielding C-capsids that contain DNA and are later transported out from the nucleus to form virions (12, 31, 32). The C-capsid structure is nearly identical to that of the virions except for the capsid-associating tegument proteins and later acquired lipid envelope (33–35). In addition to C-capsids, two dead-end capsid types, B- and A-capsids, are formed. B-capsids do not initiate DNA packaging and retain cleaved scaffold proteins (33, 34). A-capsids release scaffold protein and initiate DNA packaging but fail to retain the DNA (34). These maturation steps are summarized in Fig. 1.

**FIG 2:**
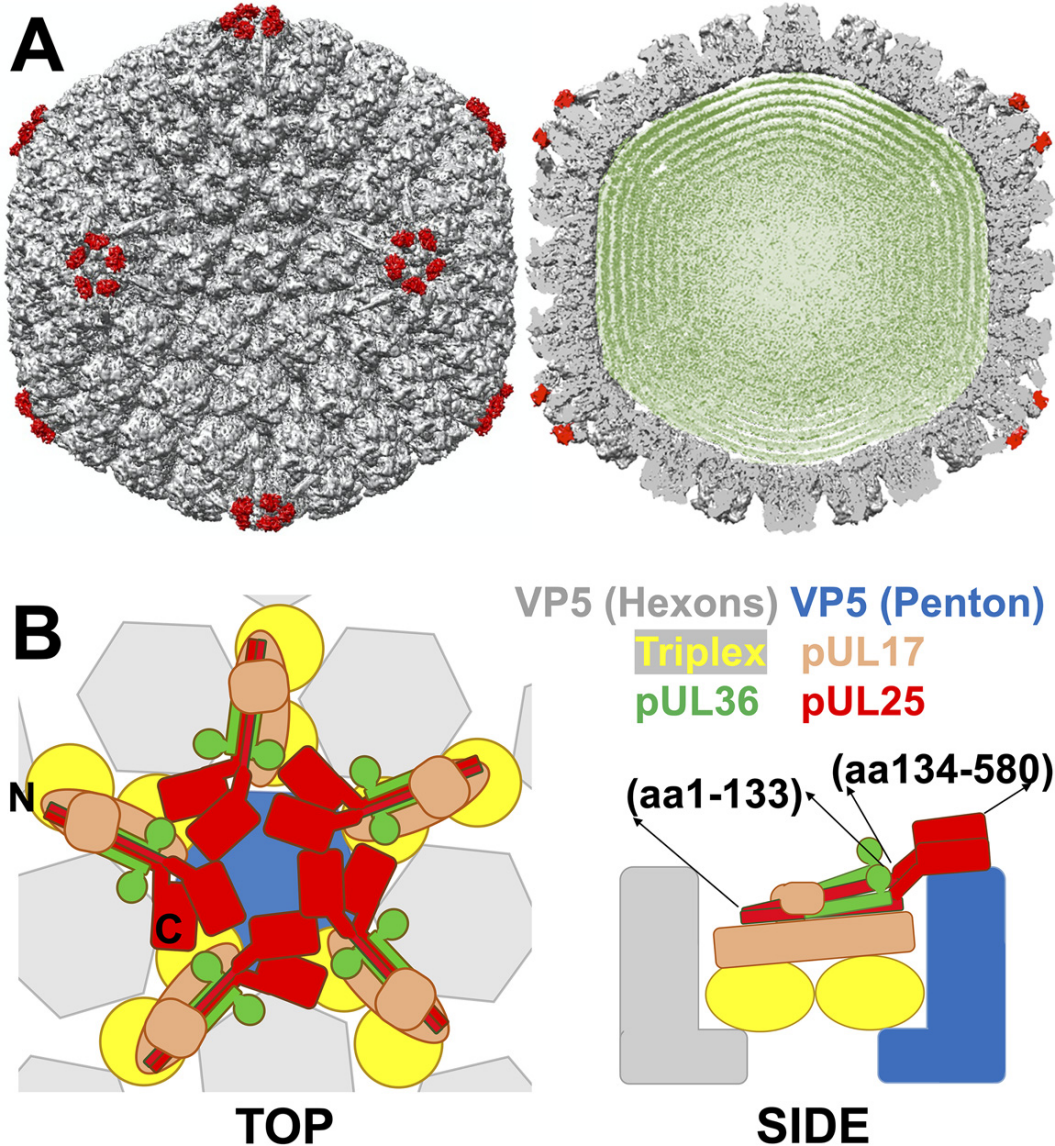
(A) 3D cryo-electron microscopy reconstruction of wild-type C-capsids imaged within intact HSV-1 virions (EMD-6386 (52)) with five copies of pUL25 (PDB 2F5U (26)) fit around each capsid penton. (B) The binding locations of pUL25 (N and C termini and the approximate amino acid positions are indicated) and other CVSC proteins are shown in the top and side view cartoons.

**FIG 3:**
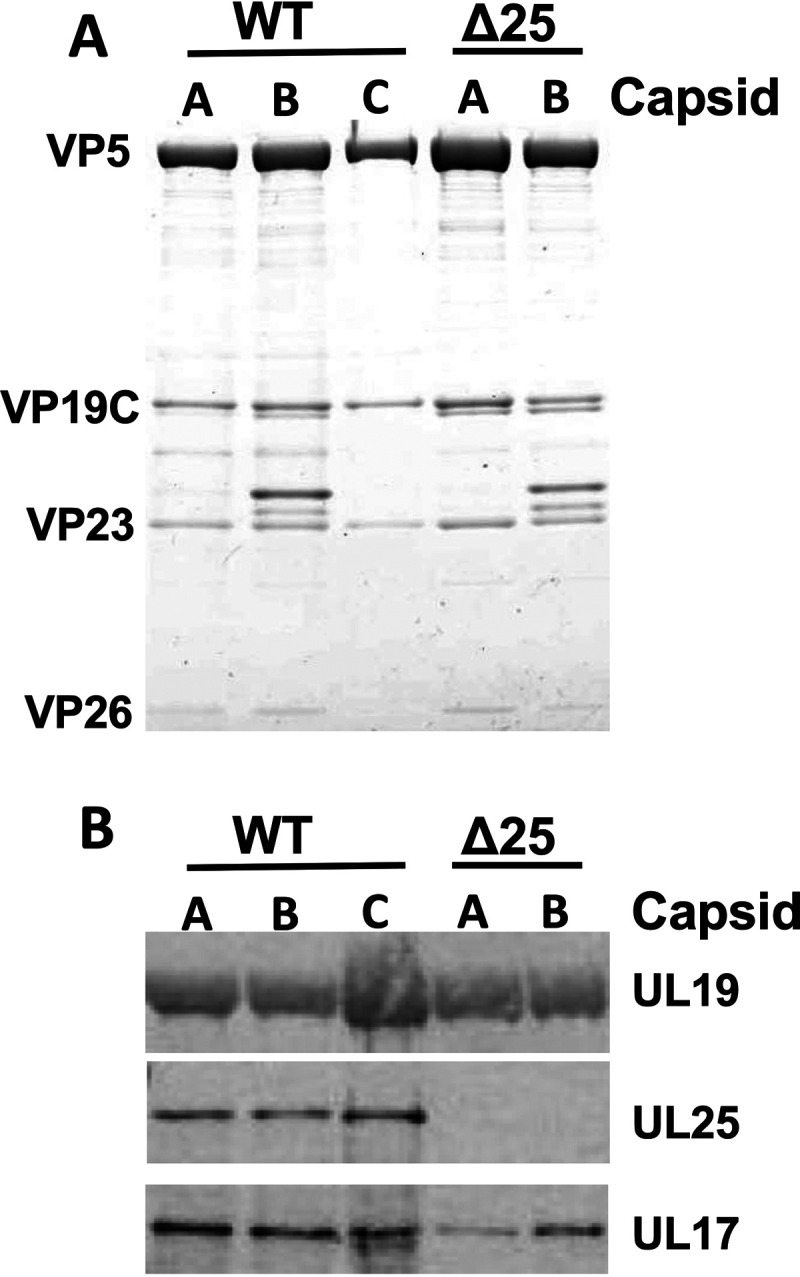
Identification of the CVSC subunits. (A) Coomassie-stained gels of WT (KOS HSV-1) and UL25-null (Δ25) capsids demonstrate approximately equal amounts of the sample according to capsid protein bands. (B) Western blots showing UL25 and UL17 on WT and UL25-null capsids. Data were adopted from our previous publication (3).

The HSV-1 capsid is mainly comprised of the major capsid protein VP5, which is organized in 11 pentameric (penton forming vertices) and 150 hexameric (hexon) capsomer subunits (36). The twelfth vertex is occupied by a portal complex forming a dodecameric ring of UL6 protein that serves as the portal channel by which DNA is packaged, and it is essential for cleavage of replicated viral DNA into the preformed capsid (37). The capsomer subunits are stabilized at all 3-fold and quasi-3-fold axes by the triplex heterotrimers, two VP23 proteins, and one VP19C protein (31) (Fig. 2). A-, B-, and C-capsids contain an equal number of major capsid proteins, triplexes, and portal proteins but differ in the abundance of the capsid vertex-specific complex (CVSC) proteins. CVSC proteins are described as minor capsid proteins and consist of a singlet of UL17 and duplexes of both UL25 and UL36, a tegument protein that might be involved in the recruitment of other tegument components to the mature capsid (37) (Fig. 2 and 3). CVSC proteins surround each of the 12 vertices (11 pentons and a portal vertex) (Fig. 2A and B). The CVSC protein number can, however, be higher at the portal (37, 38). Twelve vertices are the weakest mechanical points on the capsid due to an out-of-plane angle of the capsomer subunits inducing large lateral and radial stresses, which is unlike hexon capsomers (39, 40). This was demonstrated by thermal capsid destabilization experiments (39, 40). The C terminus of the UL25 dimer was shown to interact with the top of the VP5 penton (Fig. 2). UL25 binding around pentameric capsid vertices was found to stabilize the vertex structure, resulting in the overall increased capsid stability (3, 4, 10). A-, B- and C-capsid types reflect intermediate states of capsid maturation and display progressively increased UL25 copy number on the capsid surface that follows the progression of C > A > B. In addition, the low copy number of UL25 is found on the procapsids (12). We recently found that UL25’s function is similar to that of auxiliary proteins in dsDNA phages (e.g., phage λ), which mechanically reinforce capsids (3, 14). In phage λ, auxiliary protein gpD cements the mature capsid structure as DNA packaging nears completion (3). gpD binding to λ-capsid is timed with DNA packaging because it is facilitated by procapsid expansion that is driven by DNA pressure buildup. Before capsid expansion, gpD binding sites are not accessible (41, 42). Timed capsid reinforcement in phage λ is an essential part of the maturation process, minimizing capsid assembly errors while also supporting stable packaging of the pressurized genome (43). HSV-1 procapsid, however, does not undergo an expansion like phage λ procapsid (44). Furthermore, UL25 proteins are already associated with HSV-1 procapsid but at a significantly reduced amount compared to mature capsid (12). These observations challenged the hypothesis that the mechanical functionality of UL25 is essential for packaging pressurized DNA in herpesviruses. In this work, we observed a mechanism of UL25-mediated stepwise capsid reinforcement necessary to stably package and retain pressurized DNA.

We recently found that the gradual binding of UL25 monomers to UL25-null HSV-1 A-capsids results in increasing capsid reinforcement, suggesting that UL25 controlled mechanical capsid maturation is plausible (14). Here, by progressively deleting the length of UL25 protein bound on the surface of HSV-1 capsid (using UL25 mutants) and measuring the capsid’s strength and stiffness with atomic force microscopy (AFM), we showed which regions of UL25 affect capsid stability. By investigating the effect of differential and systematic UL25 region deletions on the stability of several maturation dead-end products of the HSV-1 capsid, which represent intermediate capsid structures in relation to the DNA packaging process, we proposed the mechanism of UL25 capsid binding before and during DNA packaging. Such mechano-genetic interrogation has not been previously attempted and was long overdue for UL25-induced capsid stabilization, which is central to herpes virion assembly.

## RESULTS AND DISCUSSION

**FIG 4:**
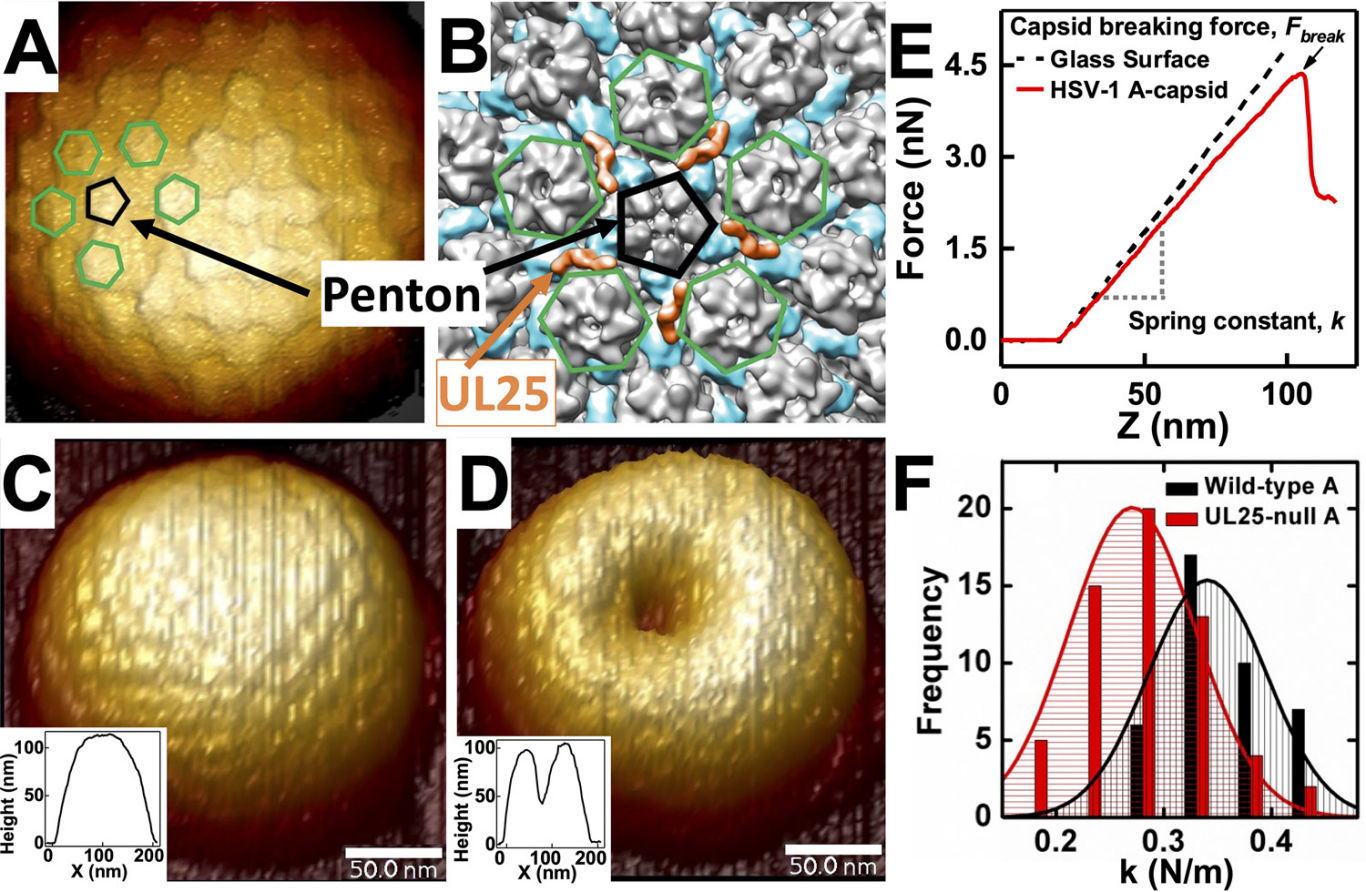
(A) Cryo-EM reconstruction (EMD-1354 (54)) is colored to indicate the positions of UL25 (orange) in relation to the penton (black pentagon outline). UL25 connects capsomers by binding with the penton and the underlying triplexes (blue on reconstruction). Neighboring hexons are outlined in green. (B) A high-resolution AFM image of HSV-1 A-capsid with a penton (black) and its five neighboring hexons (green) are indicated. (C) AFM image of intact HSV-1 A-capsid in liquid. The inset shows a topographical profile. (D) AFM image and topographical profile of the same HSV-1 A-capsid after breaking with nano-indentation. (E) Force-distance (FZ) curves for glass substrate and HSV-1 A-capsid. Capsid breaking is observed as a drop in the force curve. FZ curve allows direct measurement of *F_break_* and *k* values. The *k* values are quantified by fitting Gaussian functions to histograms of multiple measurements of unique capsids. (F) Representative histograms of spring constants, *k*, for HSV-1 WT A-capsid versus UL25-null A-capsid showing a weakening of the A-capsid with deleted UL25.

We used an AFM single-particle approach to measure the mechanical properties of viral capsids. First, individual capsids were scanned to obtain a well-resolved image (Fig. 4A to D). Fig. 4B shows the cryo-electron microscopy (cryo-EM) density and demonstrates the location of UL25 around pentons on a capsid surface. Pentameric and hexametric capsid surfaces are resolved with AFM topographical imaging in Fig. 4A. The cantilever tip was placed at the center of the capsid surface before the indentation. We recorded the force-resisting indentation when the AFM tip was brought into contact with the capsid in solution (45). The force-distance curve in Fig. 4E was linear, suggesting an elastic deformation of the HSV-1 capsid (3, 14). The slope of the force-distance curve was the spring constant *k*, which describes the stiffness and stability of the viral shell (Fig. 4F). To improve the statistics (due to the nature of a single particle experiment), multiple indentations were performed for each capsid type and histograms of spring constants for each capsid type were fitted with a Gaussian to provide the average *k* value and the standard error. At least 15 to 20 particles were measured to obtain each *k*. We also determined the maximum force at which the capsid breaks (*F_break_*) from the AFM tip indentation, which directly reflected the strength of the capsid. *F_break_* is determined as the critical force value that causes an abrupt drop in the linear force-distance curve, as shown in Fig. 4E. Following indentation, we recorded a topographical image of the capsid (Fig. 4C and D) that shows the HSV-1 capsid before and after breaking. To explore the mechanism of capsid stabilization before, during, and after DNA packaging, it was necessary to investigate the mechanics of intermediate states of capsid maturation products. The HSV-1 capsid assembly process offers a unique opportunity to accomplish this due to the ability to isolate B- and A-capsids, the dead-end products of C-capsid assembly. As mentioned above, while B- and A-capsids are not considered to be assembly intermediates because they are stably copurified with C-capsids, they directly reflect the intermediate states of capsid maturation toward C-capsid with associated DNA packaging progression (13, 46–48). High-resolution cryo-electron microscopy and tomography experiments conducted on each stable form of herpesvirus particles (29, 33, 44, 49–52) yielded 3D particle reconstructions that highlighted the structural distinctions between procapsids, B-, A-, and C-capsids, and enveloped virions. These reconstructions may serve as “snapshots” that capture the progression of capsid structure throughout the maturation, DNA packaging, and envelopment processes. Several recent studies have suggested that B-capsids may be viable intermediates awaiting DNA packaging to turn into C-capsids (33, 49). The jury is still out on whether the A- and B-capsids are true intermediates.

In our previous work, we observed that isolated wild-type (WT) B-capsids are mechanically weaker than WT A-capsids (Table 1) (3). However, UL25-null B- and A-capsids were mechanically similar but less stable than their WT counterparts (3). This difference in strength could be attributed to a reduced number of UL25 proteins bound to B-capsids (caused by fewer accessible UL25 binding sites on the capsid), weaker UL25-capsid interactions in B-capsids than in A- or C-capsids, or a combination of both scenarios. Indeed, the UL25 protein has been observed in increasing copy numbers from B- to A- to C-capsids. However, various studies reported different amounts of capsid-bound UL25 to all three types of the HSV-1 capsid, making the interpretation confounded (10, 25, 28, 37, 49, 53, 54). Determining the number of capsid-bound UL25 is difficult because, during capsid purification, a portion of CVSC proteins is lost (55). It has been proposed that because A-capsids have been exposed to DNA packaging but did not retain the DNA (13) while B-capsids have not undergone DNA packaging, the differences in UL25 binding site number and/or binding affinity between B- and A-capsids could be associated with the DNA packaging process, resulting in enhanced UL25 capsid binding interactions in A-capsids (54). In this work, we raised the question of whether the contact area that facilitates the UL25-capsid interaction was different between A and B-capsids and accounts for the difference in resulting capsid strength in addition to a potentially variable UL25 copy number between A- and B-capsids. Because angularized shells of B-, A-, and C-capsids are essentially identical in structure (20, 56), we explored the effect of varied UL25 interactions with the capsid by directly comparing measured spring constants (*k*) and breaking forces (*F_break_*) for all three types of HSV-1 capsids with various UL25 deletion mutations. It can also be noted that the scaffold protein remaining in B-capsid does not contribute to its mechanical stability (3).

**TABLE 1:** Breaking forces and stiffness of the five UL25 mutant capsids and WT capsids where WT capsids contain 580 amino acid UL25

Virus	*F*break (nN)	*k* (N/m)
**UL25-null B-capsid**	**3.0 ± 0.1**	**0.26 ± 0.01**
UL25 Δ1-50 B-capsid	3.1 ± 0.1	0.27 ± 0.01
UL25−104s B-capsid	3.9 ± 0.1	0.33 ± 0.01
UL25-212s B-capsid	3.9 ± 0.1	0.33 ± 0.01
UL25-560s B-capsid	3.9 ± 0.1	0.33 ± 0.01
UL25-577s B-capsid	3.9 ± 0.1	0.33 ± 0.01
**WT B-capsid**	**3.9 ± 0.1**	**0.33 ± 0.01**
**UL25-null A-capsid**	**3.4 ± 0.1**	**0.27 ± 0.01**
UL25 Δ1-50 A-capsid	3.4 ± 0.1	0.27 ± 0.01
UL25−104s A-capsid	4.5 ± 0.1	0.33 ± 0.01
UL25-212s A-capsid	4.8 ± 0.1	0.33 ± 0.01
UL25-560s A-capsid	5.0 ± 0.1	0.33 ± 0.01
UL25-577s A-capsid	5.3 ± 0.1	0.33 ± 0.01
**WT A-capsid**	**5.3 ± 0.1**	**0.34 ± 0.01**
UL25-577s C-capsid	5.8 ± 0.1	0.35 ± 0.01
**WT C-capsid**	**5.7 ± 0.1**	**0.35 ± 0.01**
WT C-capsid + Sp4+	5.5 ± 0.1	0.33 ± 0.01

### Distinct UL25 domains facilitate stepwise reinforcement of HSV-1 capsid.

**FIG 5:**
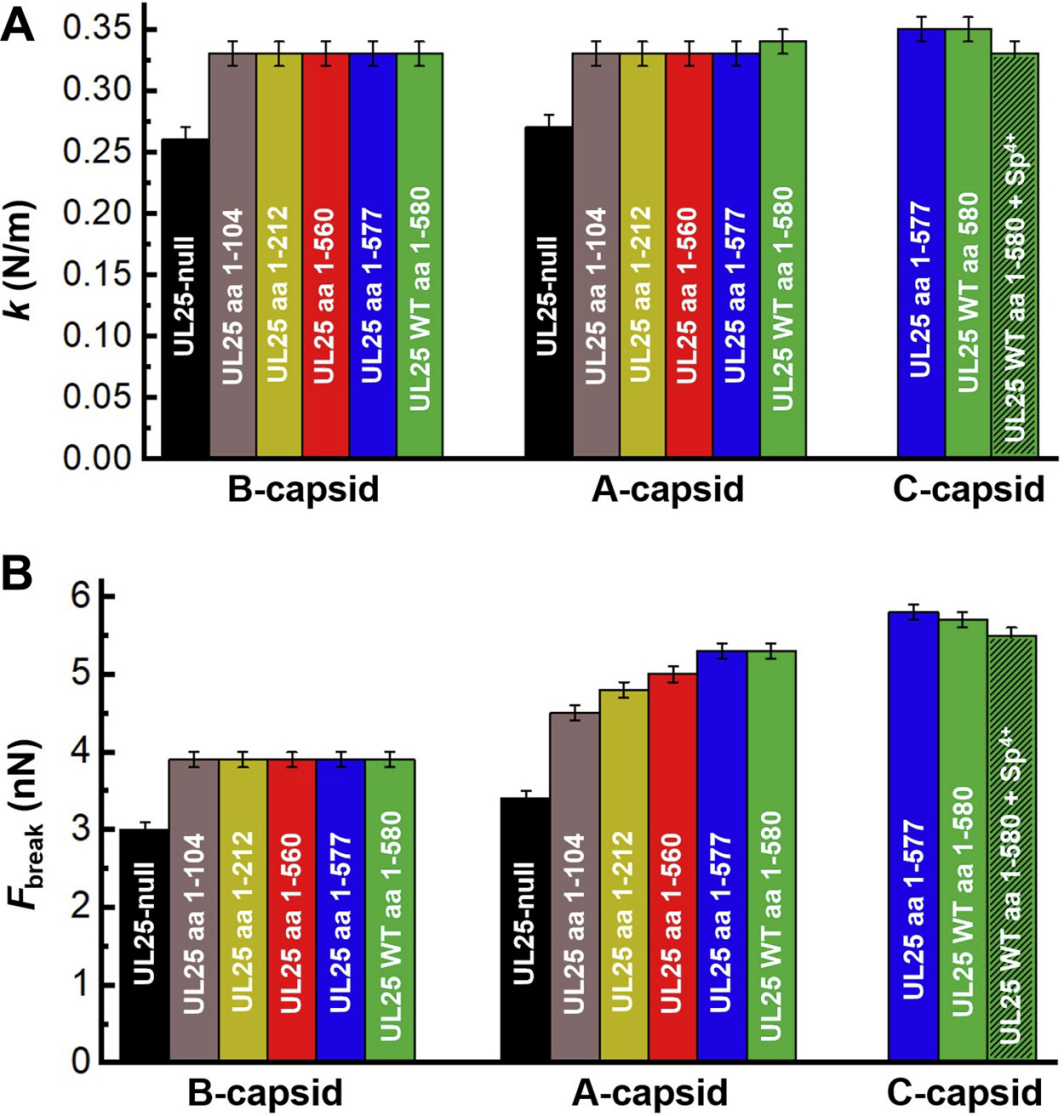
Spring constant, *k*, (Fig. 4A) and breaking force, *F_break_*, (Fig. 4B) reflect the mechanical stability of HSV-1 UL25 mutant capsids as a function of the number of amino acids (aa) in UL25 before the stop codon (s) (WT UL25 has 580 aa). For both B-capsids and A-capsids, spring constant (top) and breaking force (bottom) showed a significant increase when UL25 aa length is increased from aa 0 (UL25-null) to aa 104. For B-capsids, both breaking force and stiffness remain constant when aa length of UL25 is further increased from aa 104 to aa 580. For A-capsids, while the spring constant is unchanged for UL25 mutants with aa 104 to 580, the breaking force continued to rise with increasing aa of UL25 up to aa 577. UL25 aa 1 to 577 mutant had the same capsid stability as WT UL25 capsid with 580 aa UL25.

By comparing UL25-reinforcement of dead-end capsid maturation intermediates (B- and A-capsids), we investigated whether the DNA packaging process in A-capsids led to the exposure of extra UL25 binding sites on the capsid. Using A-capsids for our analysis allowed us to modify the UL25 interactions with the capsid through UL25 mutations, which cannot be done with fully mature C-capsids because most UL25 mutations preclude the formation of the C-capsid (18). However, it can be noted that WT A-capsids and DNA-filled C-capsids have similar mechanical properties, with C-capsids being slightly stronger (F_break_ ∼5.7 nN versus ∼5.3 nN) (Table 1). This difference may be attributed, in part, to pressurized DNA inside C-capsids, which contributes to an extra force that resists AFM tip capsid indentation as we previously observed (3). We confirmed this here by measuring *k* and *F_break_* values for C-capsids in 1 mM spermine^4+^. Polycationic spermine^4+^ ions penetrate a viral capsid and condense intracapsid DNA, which, in turn, reduces or eliminates DNA pressure (57). Indeed, the *F_break_* value for C-capsid with added 1 mM spermine^4+^ was decreased from ∼5.7 nN to ∼5.5 nN. The value of *k* was also reduced from ∼0.36 to 0.33 N/m, which was the same *k* value as for WT A-capsid (Fig. 5 and Table 1).

**FIG 6:**
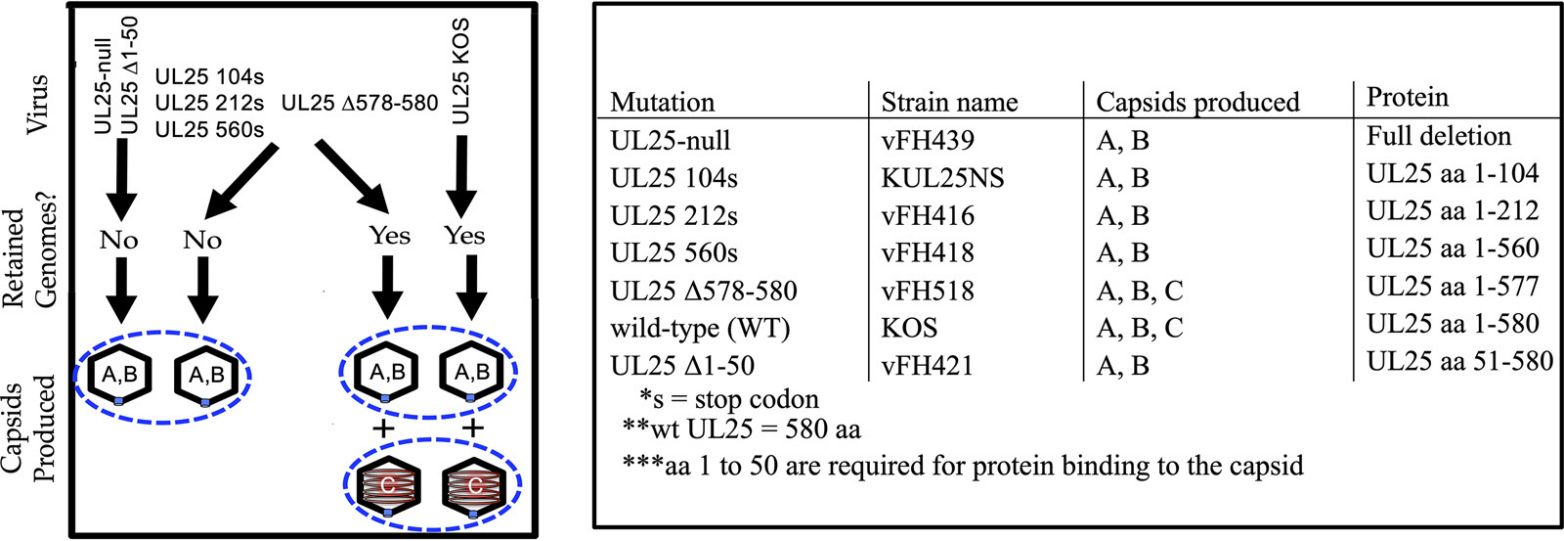
Many of the characterized UL25 mutations can be divided into three types. The first represents mutations that inhibit UL25 binding to capsids and result in a loss of infectivity. These mutations typically map to the N-terminal domain of UL25, which is essential for capsid interaction. Because these mutants do not bind capsids, their stability would be identical to UL25-null capsids. The second type of mutants binds capsids but does not retain packaged genomes. The third type consisted of a mutant that was missing the last three amino acids of UL25. This UL25 mutant Δ578-580 (UL25 aa 1 to 577) binds to capsids, and the capsids retained packaged genomes forming C-capsids but failed to produce infectious viruses. All three types of mutants were used to investigate the role of UL25 in capsid reinforcement. Coils in the C-capsids represent packaged DNA while A- and B-capsids are empty. Table 1 shows the list of HSV-1 UL25 mutants analyzed with AFM nano-indentation assay.

Many of the characterized UL25 deletion mutants of HSV-1 can be divided into three types. The first represents mutations that inhibit UL25 binding to capsids and result in a loss of infectivity. These mutations typically map to the N-terminal domain of UL25, which is essential for primary capsid binding interaction (21). Because these mutants do not bind to capsids, their mechanical stability would be identical to UL25-null capsids. The second type of mutant binds capsids but does not retain packaged genomes (17, 18). The third type of mutant, UL25 Δ578-580 (UL25 aa 1 to 577), binds capsids, and the capsids retain packaged genomes to form C-capsids but fail to produce infectious virions because capsids docked at the nuclear pore complex during infection do not eject their genome into a host nucleus (17) (Fig. 6, see the illustration along with a list of mutants). The UL25 Δ578-580 (UL25 aa 1 to 577) has the last three amino acids of UL25 truncated at the C terminus. Deletion of the three C-terminal amino acids removes one of the six unstructured loops, L6, required for DNA ejection (17). All three types of mutants were used here to investigate the role of UL25 regions on capsid reinforcement that was correlated with the ability to stably package and retain DNA (Fig. 6, see the list of mutants). SDS-PAGE analysis and Coomassie blue staining analysis of these mutants are shown elsewhere (17–19, 21). By investigating the influence of these UL25 deletion mutations on capsid strength and stiffness, we determined the specific domains of UL25 that enhanced the stability of B- and A-capsids, respectively. We used an AFM nano-indentation approach to determine the values of *F_break_* and *k* for UL25 deletion mutants of B- and A-capsids that have 0, 104, 212, 560, and 577 aa of UL25 (truncated from the C terminus where WT UL25 has 580 aa) as well as aa 51 to 580 UL25 mutant (UL25 Δ1-50, truncated from N terminus). The mechanical properties of these UL25 mutants were measured and directly compared to the WT UL25 B- and A-capsids.

First, we probed the mechanical stability of the UL25 mutant with N-terminal truncation of aa 1 to 50 of UL25 (UL25 Δ1-50), which are essential for UL25 capsid binding (18, 21). AFM measured *F_break_* and *k* showed that this mutant was mechanically identical to UL25-null B- and A-capsids, which were weaker than the WT B- and A-capsids (Table 1). This confirmed that UL25 does not bind to the capsid without the first 50 amino acids (18, 21). Next, we investigated the mechanical properties of the second group of UL25 mutants where the C-terminal truncated UL25 bind capsids but did not retain the packaged genome. These UL25 mutants are aa 1 to 104, aa 1 to 212, and aa 1 to 560 (17, 18). The breaking force and the stiffness of these UL25 mutants were compared to the UL25-null capsids as well as the WT B- and A-capsids (Fig. 5A, see *k* values; Fig. 5B, see *F_break_* values) (Table 1). The *k* and *F_break_* values for B-capsids displayed an abrupt jump when the UL25 length was increased from aa 0 to 104 with an ∼30% increase in *F_break_* and an ∼27% increase in *k*. As described above, the first 50 aa from the N terminus were required to initiate UL25 capsid binding. Interestingly, when the UL25 length was progressively increased for the UL25 mutant B-capsids from aa 104 to aa 580 (corresponding to WT UL25), the values of both *F_break_* and *k* remained constant. In contrast, Fig. 5B shows that the *F_break_* for A-capsids continued to increase with increasing UL25 aa length from aa 104 to aa 580 (corresponding to WT UL25 A-capsid). The value of *F_break_* showed an initial jump by ∼32% from 3.4 nN at 0 aa (UL25-null A-capsid) to 4.5 nN at aa 104 UL25. From aa 104 to aa 580 UL25 mutants, the *F_break_* continued to increase at an ∼18% lower rate from 4.5 nN to 5.3 nN. However, stiffness, *k*, for A-capsids remained constant at 0.33 N/m from aa 104 to aa 580 after the initial jump by ∼22% when UL25 length was increased from aa 0 to 104 (from 0.27 N/m to 0.33 N/m) (Fig. 5A). Recent cryo-EM structural analysis of the CVSC-capsid binding (29, 37) helped to provide a plausible explanation to these observations of distinctly different reinforcement behaviors between B- and A-capsids.

As described above, the capsid vertex specific complex (CVSC) consists of a UL25 dimer, a UL17 monomer, and a UL36 tegument dimer (37). Each CVSC contains a triplex-binding domain and a bi-lobed head domain (29). The triplex-binding domain is formed by two UL25 N termini and two UL36 C termini interacting with one UL17 helix, forming together a five-helix bundle that sits on top and bridges two triplexes (Ta and Tc) (Fig. 2) (37). Specifically, four helices from UL25 and UL36 interact with each other and bind on top of UL17 where UL17 deletion prevents binding of these two proteins (17, 37) (Fig. 2B and Fig. 6), while the end of N terminus of UL25 contacts the Tc triplex (37). The triplex-binding domain of UL25, which sits on top of UL17 and bridges two triplex proteins of the five-helix CVSC bundle, is well resolved in cryo-EM studies, suggesting its rigid structure (29, 37). At the same time, the bi-lobed head domain formed by the C termini of UL25 dimer was not well resolved, indicating its higher level of flexibility (29). Indeed, multiple structural studies suggested that the UL25 bi-lobed head domain makes flexible, nonidentical contact with the VP5 penton (29). The triplex-binding domain of UL25 was suggested to be within aa 1 to 133 and the head domain within aa 134 to 580 (17). Therefore, for the UL25-104s mutant (aa 1 to 104), the C-terminal truncated UL25 is rigidly bound to the triplex region of the capsid. Binding of UL25 triplex region to capsid (triplex and UL17) (37) provided significant capsid reinforcement that was reflected by a jump in the values of *k* and *F_break_* for B- and A-capsids, as shown in Fig. 5A and B. The increase in both *k* and *F_break_* for UL25-104s mutant’s B- and A-capsids compared to UL25-null capsid was similar. For B-capsid, the value of *F_break_* was increased by ∼30%, and the value of *k* was increased by ∼27%. For A-capsid, the value of *F_break_* was increased by ∼32%, and the value of *k* was increased by ∼22%. However, the addition of the UL25 head domain (UL25 mutants aa 212 to 580) (17) resulted in further reinforcement of the A-capsid but not the B-capsid. Thus, the interaction between the flexible UL25 head domain and the VP5 pentons resulted in increased *F_break_* and *k* values for A-capsids, but these interactions either do not occur or do not result in measurable changes in the values of *F_break_* and *k* in B-capsids. It is interesting to note that the N-terminal truncated mutant UL25 Δ1-50 expressed the head-region of UL25 but did not bind B- or A-capsids when the triplex-binding domain of UL25 was missing. This suggested that the head-region interactions with the capsid were weaker than that of the triplex-region interactions, and these interactions alone were not sufficient to retain UL25 bound to the capsid.

We previously established that the breaking force (*F_break_*) is a more sensitive parameter of the capsid strength compared to a spring constant value (*k*) (3). The values of *k* and *F_break_* reflect different mechanical capsid properties - stiffness versus strength. Capsid stiffness, reflected by the spring constant (*k*) measurement, was performed in a fully elastic deformation regime where the capsid was indented with the AFM tip to an extent where deformation was reversible upon its retraction. The breaking force (*F_break_*), on the contrary, was recorded when capsomer-capsomer interactions are dissociated through an irreversible AFM tip indentation and resulting in the collapse of the whole capsid, which directly reflects the strength of these interactions (Fig. 4E). Therefore, the *F_break_* parameter reflects both flexible and rigid protein-protein interactions that contribute to the overall capsid stability. The value of *k*, on the other hand, can leave flexible capsid-protein interactions undetected because they may not contribute to capsid stiffness during equilibrium indentation (3, 40). These differences in capsid mechanical properties could be reflected in the AFM measurements of the UL25 capsid mutants aa 1 to 212, aa 1 to 560, aa 1 to 577, and WT aa 580 of both B- and A-capsids (Fig. 6). As observed, flexible tethering of the head domain of the bi-lobed UL25 dimer (Fig. 2B, aa 134 to 580) to the B- and A-capsid surface did not contribute to capsid stiffness (*k*) but its binding to A-capsid VP5 penton was reflected by an increase in the A-capsid strength, as shown by the *F_break_* value (Fig. 5B).

The third type of UL25 mutant in this study was the UL25 Δ578-580 HSV-1 (capsids with aa 1 to 577 of UL25 protein) (17), which formed C-capsids that retained packaged DNA in addition to B- and A-capsids (unlike the other UL25 mutants) (Fig. 6). DNA retention in C-capsids was facilitated through capping of the extended portal complex with UL25 once DNA packaging was completed (44) (Table 1). This suggested that amino acids 560 to 577 of the UL25 head domain provided critical interactions with the portal proteins, allowing portal capping and retention of packaged DNA (17). Fig. 5 and Table 1 show that there was a continued increase in the A-capsid strength (*F_break_*) when the UL25 length was extended from 560 aa to 577 aa. UL25 aa 1 to 577 (UL25 Δ578-580) A-capsid showed the same *F_break_* (∼5.3 nN) as the WT A-capsid (Table 1). Furthermore, UL25 aa 1 to 577 C-capsid had a *F_break_* value equivalent to that of the WT C-capsid with 580 aa UL25 (*F_break_* ∼5.8 nN and *k* ∼0.35 N/m) (Table 1). It was striking to observe that the maximum mechanical capsid strength required for retention of pressurized DNA was ensured by the interactions between an essentially full-length UL25 protein with the capsid except for the last three aa of UL25 580 aa, which is instead involved in interaction with NPC, and resulted in portal uncapping and DNA ejection into cell nucleus (17)). Many UL25 residues may also be involved in UL25 folding and stability or the correct location and orientation of domains of UL25 that mediate capsid binding. This finding further supports UL25’s essential role in the mechanical capsid stabilization that was required for stable packaging and retention of the pressurized HSV-1 genome. It showed the intimate relation between molecular protein-protein interactions and mechanical information that was programmed in the structure of UL25 minor protein.

### Conclusions.

In this work, by combining genetic manipulation of UL25 and affecting its capsid binding capacity with mechanical stability measurements on resulting UL25 mutants of B-, A-, and C-capsid type dead-end intermediates, we suggested the progression of mechanical capsid maturation with regard to DNA packaging. By progressively truncating the length of UL25 that binds capsids, we found which domains of the UL25 molecule support stabilization of B-capsid (before DNA packaging) and A-capsid (where DNA packaging was initiated). Recent cryo-EM studies found that UL25 has two distinct binding domains, a triplex-binding domain (from the N-terminus) that allows for rigid capsid binding and a head domain (from the C-terminus) that displays flexible binding at the VP5 capsid penton (29, 37). We found that, after deleting the flexible head domain of UL25, the binding of the triplex/UL17-binding UL25 domain to the capsid alone provided a similar increase in strength of B- and A-capsids relative to UL25-null B- and A-capsids (Fig. 5 and Table 1, ∼30% increase in *F_break_*). This suggests that there was no principal difference in the binding affinity of the UL25 triplex-binding domain sites between B- and A-capsids. Most strikingly, however, was the observation that the head domain of UL25 did not reinforce the B-capsid while it significantly reinforced the A-capsid. This suggests that while triplex-binding sites are equally exposed in B- and A-capsids and only A-capsids expose UL25-head dimer binding sites at VP5 pentameric vertices. This structural change in A-capsids could be due to DNA pressure buildup during the DNA packaging process. The idea that DNA exerts pressure on capsid vertices, inducing changes in vertex structure, is supported by the fact that the portal complex located at one of the vertices was shown to be extended by DNA packaging pressure because it is otherwise retracted into the capsid interior in both procapsid and B-capsid of HSV-1 (37, 44). It is interesting to note that the portal remains extended in DNA-filled C-capsids with supporting intracapsid DNA pressure while it is retracted in A-capsids that fail to complete packaging, as shown in Fig. 1 (37). On the contrary, UL25 head domain binding sites at penton vertices that were exposed by DNA packaging in A-capsid remain “locked into place” even when DNA packaging is aborted. However, the portal extension mechanism also served as a gauge for retention of the packaged genome through UL25 portal capping. UL25 stabilization of other vertices does not serve this function. Combined, these observations suggest a mechanism of progressive UL25-supported capsid stabilization timed with DNA packaging. To facilitate this, UL25 capsid binding domains resemble a “hasp lock” consisting of a fixed part (triplex-binding domain) and a flexible hinge part (head domain). UL25 head domain binds to a “catch” binding site at VP5 penton vertex, which becomes either exposed or moved into a position sterically matching the location of the UL25 head domain when DNA pressure builds up in the capsid. Future structural analysis of CVSC protein attachment to the HSV-1 A-, B-, and C-capsid maturation intermediates will help to verify our interpretation of these mechanical data.

## MATERIALS AND METHODS

### Cells and viruses.

African green monkey kidney cells (Vero; CCL-81 from the American Type Culture Collection, Manassas, VA) were grown and maintained in Dulbecco’s Modified Eagle Medium (Cellgro) with 5% fetal calf serum (GeneMate) and 5% penicillin/streptomycin (Cellgro). HSV wild-type KOS (GenBank accession number JQ673480) (15, 58) and mutants UL25 104s (KUL25NS (15)), UL25-null (vFH439 (21)), UL25 212s (vFH416 (59)), UL25 560s (vFH418 (59)), UL25 Δ1-50 (vFH421 (59)), and UL25 Δ578-580 (vFH518 (17)) were described previously. Briefly, the mutants were generated by recombination of a KOS genome contained in a bacterial artificial chromosome (BAC) as previously described (18, 60) and confirmed by PCR amplification and sequencing.

### HSV-1 nuclear capsid isolation.

Vero cells were grown to confluence and infected with HSV-1 KOS strain at a multiplicity of infection of 5 PFU/cell for 20 h at 37°C. Cells were scraped into solution and centrifuged at 3500 rpm for 10 min in a JLA-16.250 rotor. The cell pellets were resuspended in PBS (1.37 M NaCl, 27 mM KCl, 43 mM Na_2_HPO_4_·7H2O, 14 mM KH_2_PO_4_), pooled, and again centrifuged at 3500 rpm for 10 min. This washed cell pellet was resuspended in 20 mM Tris buffer (pH 7.5) with protease inhibitor cocktail (Complete; Roche) and incubated on ice for 20 min to swell the cells. The swollen cells were lysed by the addition of 1.25% (vol/vol) Triton X-100 (Alfa Aesar) for 30 min on ice. Samples were centrifuged at 2000 rpm for 10 min and the resulting nuclei pellet was resuspended in a small volume of TNE (10 mM Tris, 0.5 M NaCl, 1 mM EDTA) buffer with a protease inhibitor cocktail. Nuclei were disrupted by sonication for 30 s (in 10 s intervals, iced between rounds) and large debris was cleared by brief centrifugation (maximum speed for 30 s). MgCl_2_ and DNase I were added to the supernatant to 20 mM and 100 μg/mL, respectively, and the sample was incubated at room temperature for 20 min. The supernatant was then centrifuged at 11750 × *g* for 90 s to pellet large debris and further cleaned of small debris by underlaying with a 3 mL cushion of 35% sucrose-TNE and centrifuging at 23000 rpm for 1 h. The capsid-rich pellet was resuspended in TNE + protease inhibitor cocktail then loaded onto a 20 to 50% (wt/wt) TNE sucrose gradient and centrifuged at 24,000 rpm in an SW41 rotor for 1 h. The A-, B-, and C-capsid bands were extracted by side puncture, diluted at least 3× in TNE buffer, and finally centrifuged at 24000 rpm for 1 h to pellet the capsids. Capsids were gently resuspended in TNE and stored at 4°C. The purification steps for mutant viruses were the same as described for the KOS strain.

### Atomic force microscopy.

All AFM measurements were performed on a MultiMode8 AFM with NanoScope V controller, NanoScope software, and NanoScope Analysis software (Bruker AXS Corporation, Santa Barbara, CA, USA). Images were acquired in Peak Force Tapping mode. All data (images and force-distance curves) were collected in liquid (TNE) and at room temperature unless otherwise specified. A droplet of 40 μL sample was deposited on a glass coverslip and incubated in a covered petri dish for 30 min to allow the capsids to adhere to the hydrophobic glass substrate. The details of substrate and sample preparations can be found elsewhere (45, 61). The sample was washed free of unbound capsids by pipetting 40 μL of TNE into the droplet several times, then a rectangular gold-coated Nitride probe (Olympus RC800-PSA, Tokyo, Japan) was carefully inserted into the droplet. The probe tip radius was 20 ± 5 nm, and the average stiffness of cantilevers was 0.06 N/m, determined by the thermal fluctuation method (62). The spring constant, *k*, and breaking force, *F_break_*, for a viral particle were obtained using the indentation measurement (directly after AFM cantilever calibration on the glass substrate) with at least 15 to 20 unique particles measured for each reported value. Spring constant measurements were made by applying a gentle force to the center of each capsid, and the details of the *k* calculation are described elsewhere (45). The breaking force was measured by applying a much larger force to the capsid, resulting in capsid mechanical failure at a particular force measured experimentally by a sudden sharp drop in the force-distance curve. Measurements were performed in HSV-1 storage buffer TNE.
